# Performance evaluation of biological safety cabinets: a real-world analysis

**DOI:** 10.3389/fbioe.2026.1801944

**Published:** 2026-05-13

**Authors:** Tao Song, Shaohua Yin, Zhenlin Liu, Xiaoqi Xue, Yilin Li, Yong Li, Xingshuo Zhang, Xiangyun Qiao, Chenglin Wu, Han Zhang, Rongxi Jia, Xiaoming Liu, Zhe Sun, Wei Yao, Bo Zhang, Feng Xu, Yun Tian, Jin Tian

**Affiliations:** 1 Department of Medical Engineering, Peking University Third Hospital, Beijing, China; 2 Department of Orthopedics, Peking University Third Hospital, Beijing, China

**Keywords:** biological safety cabinet, cross-sectional study, laboratory safety, marginally qualified performance, medical and health institution

## Abstract

**Introduction:**

Biological safety cabinets (BSCs) are essential for protecting personnel, samples, and the environment in clinical and research laboratories. Evidence on the real-world performance of Class II BSCs in China remains limited, particularly regarding differences between domestic and imported equipment.

**Methods:**

We conducted a cross-sectional evaluation of 360 Class II BSCs in Beijing using 1,803 performance testing records from 2018 to 2023. Performance indicators, including noise, illumination, cleanliness, inflow velocity, downflow velocity, HEPA filter integrity, and airflow smoke pattern were measured according to JG 170-2005, YY 0569-2011 and SN/T 3901–2014 standards. BSCs were assessed and classified as fully qualified performance (FQP) if the indicator met the standard without equaling the threshold, marginally qualified performance (MQP) if the indicator equaled the threshold, or unqualified if the standard was not met. Qualified rates were compared by manufacturing region, hospital level, using sector, and service life using *χ* ^*2*^ tests and non-parametric analyses.

**Results:**

Among 360 Class II BSCs, the median service life was 7 years (IQR 5–10). The overall qualified rate was 70.4%, with no difference between domestic and imported BSCs (71.3% *vs*. 69.2%; *χ* ^*2*^=0.909, *P*=0.340). Domestic BSCs showed higher FQP rate (55.4% *vs*. 40.5%; *χ* ^*2*^=11.625, *P*=0.0007) and lower MQP rate (15.8% *vs*. 28.7%; *χ* ^*2*^=15.087, *P*=0.0001), with similar pattern for noise, inflow velocity, and downflow velocity. The inflow velocity, downflow velocity, and illumination showed relatively lower qualified rates, with only inflow velocity having a higher qualified rate in domestic BSCs (85.5% *vs*. 80.9%; *χ* ^*2*^=6.845, *P*=0.009). Imported BSCs performed slightly better in several key indicators, including inflow velocity, downflow velocity, and HEPA filter integrity, although differences were not statistically significant.

**Discussion:**

These findings suggest that BSC reliability reflects an interplay between manufacturing-related characteristics and in-use performance, and that the MQP helps identify BSCs operating near minimum safety margins for proactive monitoring and timely intervention.

## Introduction

Biological safety cabinets (BSCs) are widely used as the primary engineering control to protect personnel, experimental materials, and the surrounding environment when handling infectious microorganisms, hazardous biological agents, and toxic substances in medical and health institutions (Pawar et al., 2021; World Health Organization, 2020b). By integrating inflow and downflow velocities with high-efficiency particulate air (HEPA) filtration, BSCs effectively limit aerosol dispersion and environmental contamination, playing a critical role in infection prevention, laboratory-acquired infection mitigation, and occupational health protection (American National Standards Institute, 2025). According to design principles and protective functions, BSCs are classified into classes I, II, and III, with Class II BSCs being the most widely deployed globally due to their balanced protection (American National Standards Institute, 2025; Anon, 2004).

International biosafety guidelines issued by the World Health Organization, the American National Standards Institute, and the British Standards Institution indicate that the protective efficacy of BSCs depends not only on their structural design but also on real-world operational performance (American National Standards Institute, 2025; British Standards Institution, 2000; World Health Organization, 2020b). Critical performance parameters including inflow velocity, downflow velocity, HEPA filter integrity, airflow smoke pattern, noise, and illumination must meet stringent technical specifications to ensure sustained biosafety (Bozkurt & ÖZnur, 2023). Laboratory incidents have shown that compromised BSC performance, such as HEPA filter leakage (Jagtap, Badge, Kohale and Wankhade, 2023), abnormal airflow patterns, or failures in cabinet airtightness and negative-pressure systems (Parks, Hookway, Kojima and Bennett, 2022; Permana, Agharid and Wang, 2025), may result in the release of infectious aerosols, leading to laboratory-acquired infections and environmental exposure (Jagtap et al., 2023). Regular performance testing, certification, and preventive maintenance of BSCs are mandatory in many high-income countries, supported by standardized testing protocols such as NSF/ANSI 49 in the United States and EN 12469 in Europe (American National Standards Institute, 2025; British Standards Institution, 2000). These frameworks underscore that biosafety is a dynamic process that depends on continuous verification rather than one-time equipment installation (National Science Foundation, 2025).

In China, the development and regulation of BSCs have progressed rapidly but unevenly. Prior to 2003, BSC deployment was limited, domestic manufacturing capacity was scarce, and national technical standards were lacking. Laboratory-associated outbreaks of severe acute respiratory syndrome between 2003 and 2004 prompted heightened national awareness of laboratory biosafety risks (Dennis and Normile, 2004), accelerating biosafety infrastructure development, mandating BSC installation, and incorporating BSCs into Class III medical equipment management under standards such as JG 170 (Ministry of Construction of the People’s Republic of China., 2005) and YY 0569 (China Food and Drug Administration, 2005). Despite these advances, challenges remained in standardized performance evaluation, routine certification practices, and long-term maintenance strategies compared with established international systems (World Health Organization, 2020a). Existing studies comparing domestic and imported BSC performance remain limited, mainly focusing on a single performance indicator, a small sample, or an individual laboratory. Few studies have proposed evaluative metrics capable of capturing marginal or early-stage performance degradation, which may precede outright failure yet pose meaningful biosafety risks. To address this gap, the concept of marginally qualified performance (MQP) was introduced, defined as performance parameters that meet but do not substantially exceed threshold requirements, indicating operation near minimum acceptable safety margins. In real-world practice, MQP reflects reduced safety margins or early-stage performance degradation, helping laboratory personnel and managers identify BSCs that, while still technically qualified, may benefit from closer monitoring, preventive maintenance, or timely replacement before outright failure occurs.

Beyond laboratory safety, the performance qualification of BSCs has broader socio-economic and industrial implications. High-performing BSCs ensure the uninterrupted operation of clinical laboratories, biopharmaceutical production, and vaccine research, which are critical for public health preparedness and epidemic response. Marginally performing BSCs can result in laboratory shutdowns, increased maintenance costs, and delayed research outputs, affecting healthcare delivery and industrial productivity. Furthermore, domestic manufacturing and certification of reliable BSCs support the local biomedical equipment industry, reduce dependence on imports, foster technological innovation, and generate economic value. Evaluating BSC performance, therefore, links technical standards to operational efficiency, public health outcomes, and broader societal and economic benefits.

The objective of this study was to compare MQP performance differences between domestic and imported Class II BSCs using the latest testing data from 52 hospitals in Beijing from 2018 to 2023 and explore factors associated with qualified performance.

## Methods

### Study design and data source

We conducted a cross-sectional study on Class II BSCs in Beijing, northern China, using data from routine annual performance testing between May 2018 and December 2023. Performance testing was undertaken across 52 general hospitals, including 65 clinical laboratories and 30 research sectors, spanning 13 administrative districts. BSCs and participating hospitals were eligible if (1) testing was performed by trained technologists from an authorized public testing institution, (2) the BSCs were Class II with valid medical equipment registration certificates, (3) the equipment was installed and operational at local study sites during the testing period, and (4) the hospitals agreed to comply with the study protocol.

Of 1,835 initial testing records, three were excluded due to missing key performance indicators, leaving 1,803 BSC records for the final analysis.

### Performance indicators and measurements

We evaluated seven BSC performance indicators, namely, noise, illumination, cleanliness, inflow velocity, downflow velocity, HEPA filter integrity, and airflow smoke pattern, and the measurements were conducted following national standards (JG 170–2005, YY 0569–2011, and SN/T 3901–2014) (China Food and Drug Administration, 2011; General Administration of Quality Supervision, 2014; Ministry of Construction of the People’s Republic of China, 2005).

A detailed measurement protocol is provided in the *Supplementary Methods*. In brief, noise was measured 300 mm in front of the BSC and 380 mm above the workbench using an A-weighted sound level meter, and background noise was recorded with the fan switched off for correction. Noise was considered qualified if it was 67 dB or lower. Illumination was measured along the centerline of the work surface at points spaced no more than 300 mm apart and at least 150 mm away from interior walls, and illuminance was considered qualified if it reached at least 650 lx. Cleanliness was assessed after the BSC had operated for 10 min under normal working conditions, and particle counts were measured 200 mm above the workbench and 100 mm from the interior surfaces or the front sash; compliance with ISO Class 5 air cleanliness standards indicated qualified performance. Inflow velocity was measured at two horizontal rows corresponding to 25% and 75% of the sash height using an anemometer, and a velocity of 0.50 m/s or higher was considered qualified. Downflow velocity was measured in a square grid 150 mm from the interior walls or front sash and 100 mm above the work surface, and velocities ranging from 0.25 m/s to 0.50 m/s were considered qualified. HEPA filter integrity was assessed using aerosol photometry downstream of both the supply and exhaust filters, with leakage of 0.01% or less for Type-A BSCs, along with leakage of 0.01% for the supply filter and 0.005% for the exhaust filter in Type-B BSCs considered qualified. Airflow smoke patterns were visualized at predefined points to assess downflow uniformity, airflow at the edges, and tightness of the operating window, with uniform downward airflow without vortices or air escape considered qualified.

MQP was defined for indicators that exactly met the threshold values, representing operation at the minimum acceptable safety limits. For example, noise of exactly 67 dB, illuminance of exactly 650 lx, inflow velocity of exactly 0.50 m/s, or downflow velocity at the lower or upper limit was recorded as MQP. Similarly, HEPA filter leakage or airflow deviations at their threshold limits were considered MQP. An overall MQP metric was generated whenever any individual indicator reached its marginally qualified limit. Fully qualified performance (FQP) was defined as all indicators meeting threshold values without reaching their marginally qualified limits.

### Covariates

We collected hospital- and equipment-related information from the medical equipment evaluation system, including testing year (2018–2023), hospital level (secondary or tertiary), sector (clinical laboratory or research sector), manufacturing region (domestic or imported), and BSC type (A2 or B2). Service life was calculated as the interval between the testing date and the manufacturing date (in years) and categorized as less than 5 years, 5–10 years, or 10 years or above.

### Statistical analysis

Categorical variables were presented as frequencies and percentages and compared using the chi-squared test or Fisher’s exact test. The normality of continuous variables was tested using the Shapiro–Wilk test. Normally distributed variables were described as the mean ± standard deviation and compared with the t-test or one-way ANOVA, and skewed-distributed variables were described as the median (interquartile range, IQR) and compared with the Mann–Whitney U test or the Kruskal–Wallis test, as appropriate. Multivariate logistic regression was performed to estimate adjusted odds ratios (ORs) and 95% confidence intervals (CIs) for factors associated with MQP and FQP. Analyses were further stratified by hospital- and equipment-related characteristics. A two-sided *p* < 0.015 was considered statistically significant after Bonferroni adjustment. All analyses were conducted using SAS software (version 9.4; SAS Institute Inc.).

## Results

**FIGURE 1:**
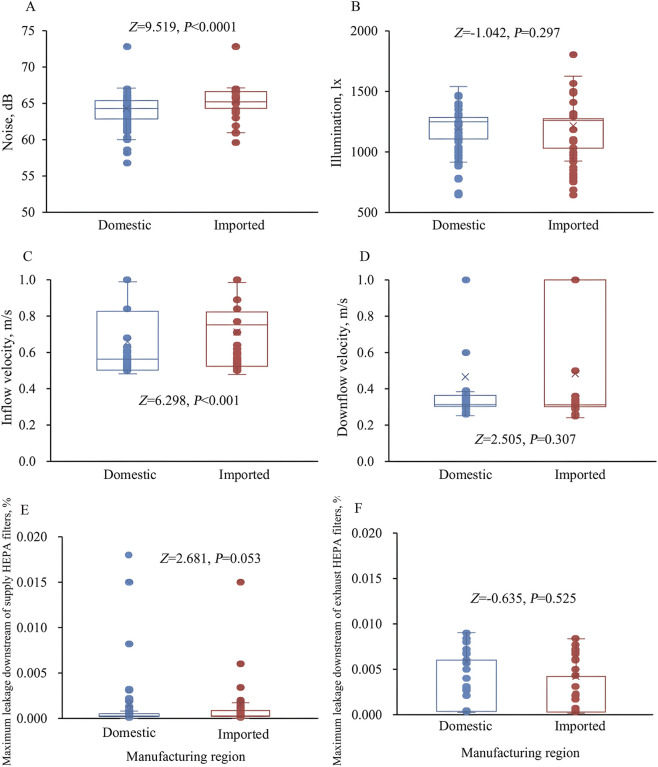
Distributions of performance indicators in domestic and imported biological safety cabinets, including **(A)** noise, **(B)** illumination, **(C)** inflow velocity, **(D)** downflow velocity, **(E)** maximum leakage downstream of the supply HEPA filters, and **(F)** maximum leakage downstream of the exhaust HEPA filters.

A total of 360 Class II BSCs with 1,803 performance testing records were included, with a median service life of 7 years (IQR 5–10). The overall qualified rate was 70.4%, with domestic BSCs at 71.3% and imported BSCs at 69.2% (*χ* ^*2*^ = 0.909, *p* = 0.340) (Table 1). Among individual performance indicators, inflow velocity, downflow velocity, and illumination showed relatively lower qualified rates, with only inflow velocity differing by manufacturing region: 85.5% for domestic *versus* 80.9% for imported BSCs (*χ* ^*2*^ = 6.845, *p* = 0.009). Analysis of continuous measurements showed that domestic BSCs had lower median noise (64.2 dB vs. 65.1 dB; *Z* = 9.519, *p* < 0.0001) and lower median inflow velocity (0.58 m/s vs. 0.77 m/s; *Z* = 6.298, *p* < 0.001) than imported BSCs (Figure 1).

**TABLE 1:** Performance of biological safety cabinets by manufacturing region^a^.

Performance indicator	Overall qualified^b^	Qualified performance	
		FQP^c^	MQP^c^
Total			
Domestic	774 (71.27)	602 (55.43)	172 (15.84)
Imported	496 (69.18)	290 (40.45)	206 (28.73)
Noise			
Domestic	1,029 (94.75)	953 (87.75)	76 (7.00)
Imported	691 (96.37)	567 (79.08)	124 (17.29)
Illumination			
Domestic	930 (85.64)	930 (85.64)	0
Imported	610 (85.08)	610 (85.08)	0
Cleanliness			
Domestic	1,086 (100.00)	1,047 (96.41)	39 (3.59)
Imported	717 (100.00)	677 (94.42)	40 (5.58)
Inflow velocity			
Domestic	929 (85.54)	799 (73.57)	130 (11.97)
Imported	580 (80.89)	542 (75.59)	38 (5.30)
Downflow velocity			
Domestic	868 (79.93)	838 (77.16)	30 (2.76)
Imported	558 (77.82)	476 (66.39)	82 (11.44)
HEPA filter integrity			
Domestic	962 (88.58)	962 (88.58)	0
Imported	625 (87.17)	625 (87.17)	0
Airflow smoke pattern			
Domestic	1,086 (100.00)	984 (90.61)	102 (9.39)
Imported	717 (100.00)	626 (87.31)	91 (12.69)

FQP, fully qualified performance; MQP, marginally qualified performance.

^a^ Data are presented as number (percentage).

^b^ Overall qualified indicates that the indicator met the qualification criteria, including both fully qualified and marginally qualified results.

^c^ Fully qualified indicates that indicator met qualification criteria without reaching marginal limits, whereas marginally qualified indicates that the indicator exactly equaled the corresponding qualification limit values.

When performance was categorized into FQP and MQP, domestic BSCs showed higher FQP rates (55.4% vs. 40.5%; *χ* ^*2*^ = 11.625, *p* = 0.0007) and lower MQP rates (15.8% vs. 28.7%; *χ* ^*2*^ = 15.087, *p* = 0.0001). The indicator-specific analyses of MQP showed similar patterns: for noise, 7.0% vs. 17.3% (*χ* ^*2*^ = 22.157, *p* < 0.0001); for inflow velocity, 5.3% vs. 11.9% (*χ* ^*2*^ = 26.125, *p* < 0.0001); and for downflow velocity, 2.8% vs. 11.4% (*χ* ^*2*^ = 33.283, *p* < 0.0001) in domestic *versus* imported BSCs (Table 1).

**FIGURE 2:**
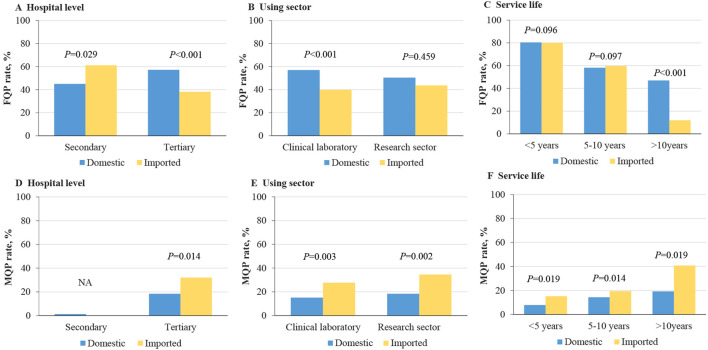
Fully qualified and marginally qualified rates stratified by hospital level, using sector, and service life, with fully qualified rates by **(A)** hospital level, **(B)** using sector, and **(C)** service life, and marginally qualified rates by **(D)** hospital level, **(E)** using sector, and **(F)** service life.

Stratification by BSC type showed consistent patterns (Table 2). Among Type-A2 BSCs, domestic BSCs had higher FQP rates (58.8% vs. 42.4%; *χ* ^*2*^ = 13.215, *p* < 0.001) and lower MQP rates for downflow velocity (2.5% vs. 12.5%; *χ* ^*2*^ = 39.921, *p* < 0.0001) and noise (8.5% vs. 18.9%; *χ* ^*2*^ = 17.226, *p* < 0.0001). For type-B2 BSCs, domestic BSCs also had higher FQP rates (57.2% vs. 38.1%; *χ* ^*2*^ = 10.083, *p* = 0.002), while MQP analysis showed a higher MQP rate for inflow velocity among domestic BSCs (27.7% vs. 0; *p* < 0.0001). Similar trends were observed when stratified by hospital level, sector, and service life (Figure 2). FQP rates were higher for domestic BSCs in tertiary hospitals and clinical laboratories and for BSCs with service life exceeding 10 years, whereas MQP rates were lower across tertiary hospitals, all using sectors, and for BSCs with 5–10 years of service.

**TABLE 2:** Performance of biological safety cabinets by manufacturing region and cabinet type^a^.

Performance indicator	Type A2		Type B2	
	FQP^b^	MQP^b^	FQP^b^	MQP^b^
Total				
Domestic	524 (58.81)	164 (18.41)	78 (40.00)	8 (4.10)
Imported	278 (42.44)	206 (31.45)	12 (19.35)	0
Noise				
Domestic	770 (86.42)	76 (8.53)	183 (93.85)	0
Imported	509 (77.71)	124 (18.93)	58 (93.55)	0
Illumination				
Domestic	778 (87.32)	0	152 (77.95)	0
Imported	554 (84.58)	0	56 (90.32)	0
Cleanliness				
Domestic	858 (96.30)	33 (3.70)	189 (96.92)	6 (3.08)
Imported	633 (96.64)	22 (3.36)	44 (70.97)	18 (29.03)
Inflow velocity				
Domestic	672 (75.42)	76 (8.53)	127 (65.13)	54 (27.69)
Imported	526 (80.31)	38 (5.8)	16 (25.81)	0
Downflow velocity				
Domestic	702 (78.79)	22 (2.47)	136 (69.74)	8 (4.10)
Imported	448 (68.40)	82 (12.52)	28 (45.16)	0
HEPA filter integrity				
Domestic	827 (92.82)	0	135 (69.23)	0
Imported	605 (92.37)	0	20 (32.26)	0
Airflow smoke pattern				
Domestic	797 (89.45)	94 (10.55)	187 (95.90)	8 (4.10)
Imported	594 (90.69)	61 (9.31)	32 (51.61)	30 (48.39)

FQP, fully qualified performance; MQP, marginally qualified performance.

^a^ Data are presented as number (percentage).

^b^ Fully qualified indicates that the indicator met qualification criteria without reaching marginal limits, whereas marginally qualified indicates that the indicator exactly equaled the corresponding qualification limit values.

**FIGURE 3:**
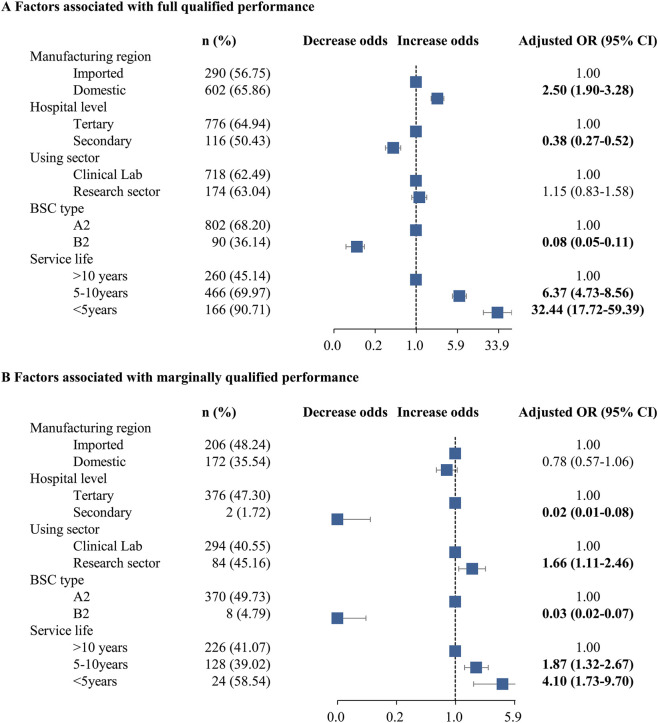
Hospital- and equipment-related factors associated with **(A)** fully qualified performance and **(B)** marginally qualified performance.

Multivariate analysis of hospital- and equipment-related factors showed that several characteristics were associated with FQP and MQP (Figure 3). Domestic manufacture was positively associated with higher FQP rates (adjusted odds ratio [aOR]: 2.50, 95% CI: 1.90–3.28). Shorter service life was also associated with higher FQP rates, with aORs of 32.44 (95% CI: 17.72–59.39) for service life <5 years and 6.37 (95% CI: 4.73–8.56) for 5–10 years. In contrast, secondary hospitals (aOR: 0.38, 95% CI: 0.27–0.52) and type-B2 BSCs (aOR: 0.08, 95% CI: 0.05–0.11) were associated with lower FQP rates. For MQP, the research sector was associated with higher MQP rates (aOR: 1.66, 95% CI: 1.11–2.46), and shorter service life showed a modest positive association (aOR: 4.10, 95% CI: 1.73–9.70 for service life <5 years; aOR: 1.87, 95% CI: 1.32–2.67 for 5–10 years). Secondary hospitals (aOR: 0.02, 95% CI: 0.01–0.08) and type-B2 BSCs (aOR: 0.03, 95% CI: 0.02–0.07) were associated with lower MQP rates.

## Discussion

In this cross-sectional study of 360 Class II BSCs assessed through 1,803 performance testing records, the overall qualified rate was similar between domestic and imported BSCs, yet domestic BSCs showed a higher FQP rate and a lower MQP rate. Indicator-specific analyses indicated that domestic BSCs had higher FQP rates for inflow velocity and lower MQP rates for noise, inflow velocity, and downflow velocity. Key performance indicators such as inflow velocity, downflow velocity, and HEPA filter integrity showed slightly better performance in imported BSCs, although the differences were not statistically significant. These findings highlight the practical relevance of the MQP concept as it identifies cabinets operating near the edge of acceptable safety limits, allowing laboratory managers to implement targeted monitoring, preventive maintenance, and timely replacement before performance deteriorates further. Overall, domestic BSCs are more likely to maintain consistent operational performance in routine use, providing insights for improving laboratory biosafety management and informing broader biosafety and biosecurity practices.

Global and national studies have reported substantial variation in Class II BSC performance. In Turkey, an empirical study of 160 BSCs in public health laboratories found an overall qualified rate above 77%, with failures mainly due to inflow and downflow velocity deviations, HEPA filter leakage, and airflow pattern imbalances (Bozkurt & ÖZnur, 2023). A multicenter assessment across seven Asia–Pacific countries reported that approximately 70% of Class II BSCs met performance qualification criteria, with the remainder performing poorly due to design, installation, or operational issues (Biosecurity Challenges of the Global Expansion of High-Containment Biological Laboratories, 2011). In Thailand, Cambodia, Laos, and Vietnam, performance-qualified rates ranged from 30% to 50%, indicating widespread challenges in maintaining equipment standards (Whistler, Kaewpan, and Blacksell, 2016). In China, a study in northern veterinary research institutes found an overall qualified rate of only 17.5% (Ma et al., 2022). Our findings of a 70.4% overall qualification rate in Beijing are broadly comparable to those reported in the Asia–Pacific region yet underscore ongoing variability and the need to strengthen routine monitoring.

The observation that domestic BSCs showed higher FQP rates and lower MQP rates may reflect their integration with local operational practices, including installation, calibration, and maintenance by local personnel familiar with equipment specifications and laboratory workflows, which supports stable performance across key indicators such as noise, airflow velocity, and HEPA filter integrity. In contrast, imported BSCs may perform closer to qualification thresholds due to variation in user training, operational habits, or adaptation to local environmental conditions. Similar findings have been reported in other studies emphasizing the impact of operational practices on BSC performance rather than the intrinsic quality of the equipment (World Health Organization, 2020b). Despite this, certain critical parameters, including inflow velocity, downflow velocity, and HEPA filter integrity, were slightly higher in imported cabinets, consistent with previous reports such as the study in Wenzhou, China, where imported Class II BSCs had higher qualified rates for cleanliness, airflow pattern, inflow velocity, and downflow velocity (Yu, Ni, Xu and Chen, 2010). These differences are likely attributable to advanced airflow control and filtration technologies incorporated in imported cabinets, such as independent supply and exhaust blowers with real-time airflow monitoring and high-efficiency H14 HEPA filters (>99.995% at the most penetrating particle size), negative-pressure plenums, and precision-machined components, which minimize leakage and maintain stable airflow. Strengthening the adoption of similar design and manufacturing practices domestically, along with rigorous certification, may help further improve the overall performance consistency of BSCs (Taylor et al., 2019; World Health Organization, 2020a).

Our findings found that individual performance indicators, including inflow velocity, downflow velocity, and illumination, showed relatively lower qualified rates, which is consistent with prior studies emphasizing the need for regular inspection and maintenance of HEPA filters and exhaust blowers to increase the qualified rate of inflow velocity and downflow velocity (Tan et al., 2013; Wen et al., 2008). Proper laboratory placement, away from drafts, convection currents, and traffic, also improves airflow stability and reduces contamination risk, and the National Science Foundation recommended that the BSCs should be placed in the laboratory away from drafts, convection currents, apparent airflow, and traffic paths (Gilpin and Powitz, 2020; NUAIR, 1979). Operator behavior, working habits, and hours of operation further affect HEPA filter dust accumulation and airflow patterns (Chuang, 2025; Parks et al., 2022; Wayne State University, 2024). The fragile and precise structure of HEPA filters makes them susceptible to blockage or damage, which can reduce inflow and downflow velocities if not replaced in a timely manner. A study from China investigated BSC performance and maintenance practices, revealing that few cabinets underwent HEPA filter replacement due to high costs and limited awareness of proper maintenance (Pan, 2012) and that most institutions relied on simpler measures, such as cleaning the working environment or the BSC itself.

Our study showed that BSCs with longer service life had lower qualified rates, reflecting cumulative wear on blowers, seals, and filters, as well as gradual loss of structural integrity, which can compromise directional airflow and containment. This is consistent with previous evidence, including a Chinese investigation of 187 Class II BSCs conducted by the National Institutes for Food and Drug Control, reporting an unqualified rate of 63% among cabinets with a service life of approximately 7 years, indicating substantial performance degradation with prolonged use (Wang et al., 2019). Although BSCs can typically remain operational for up to 15 years under ideal conditions, frequent use and challenging operating environments can accelerate performance decline (Clark et al., 1990). Differences were also observed by hospital level and sector, with secondary hospitals and clinical laboratories showing lower qualified rates than tertiary hospitals and research settings, suggesting that operational intensity, preventive maintenance, and available resources may influence long-term performance. These findings underscore the importance of incorporating service life into maintenance planning and considering timely renewal or upgrade of BSCs approaching the end of their effective operational period.

This study has several strengths. First, it is the first comprehensive assessment of BSC across domestic and imported equipment in a major Chinese city, providing evidence for public health and laboratory management. Second, the study was based on a well-organized, equipment-based evaluation system with quality control measures to ensure accurate enrollment and measurement of performance indicators. Third, the use of the MQP concept, an underused descriptive approach in epidemiology (Arora et al., 2013), allowed identification of cabinets operating at or near threshold limits, highlighting early-stage performance degradation or reduced safety margins. This enables laboratory managers to prioritize monitoring, preventive maintenance, or timely intervention, providing both a statistical perspective and practical guidance for enhancing biosafety. This study also has limitations. Detailed information on maintenance records or specific exhaust duct characteristics (e.g., diameter and length) was not collected, which might have influenced performance interpretation. The small sample sizes in some stratified analyses may have introduced sampling variability, potentially affecting the precision of multivariate regression estimates and making coefficients from sparsely represented strata less stable, which could limit the generalizability of the findings to other BSCs or laboratory settings. Finally, because participating institutions are more likely to operate highly configured BSCs, the generalizability of our findings to cabinets with lower or moderate configurations in low- and middle-income regions may be limited.

## Conclusion

In this study, we showed that the overall performance qualification rate was similar between domestic and imported BSCs. Domestic BSCs showed higher FQP rates and lower MQP rates, whereas imported BSCs showed slightly better performance in key indicators. These findings suggested that BSC reliability reflected an interplay between manufacturing characteristics and operational use and that the MQP framework provides additional insights into cabinets operating near minimum safety margins.

## Data Availability

The original contributions presented in the study are included in the article/Supplementary Material, further inquiries can be directed to the corresponding authors.
