# Supplementary material for: Performance evaluation of biological safety cabinets: a real-world analysis
Source: Front Bioeng Biotechnol. 2026 May 13;14:1801944. doi: 10.3389/fbioe.2026.1801944 (PMC13212249; doi:10.3389/fbioe.2026.1801944)
Supplement: Supplementary file 1 [file Supplementaryfile1.docx]

**Supplementary Methods**

Seven performance indicators for biological safety cabinets, including noise, illumination, cleanliness, inflow velocity, downflow velocity, HEPA filter integrity, and airflow smoke pattern were conducted following national standards (JG 170–2005, YY 0569–2011, SN/T 3901–2014) (China Food and Drug Administration., 2011; General Administration of Quality Supervision, 2014; Ministry of Construction of the People's Republic of China., 2005), and the measurements as follows:

**Noise** was measured under standard operating conditions at a position 300 mm in front of the BSC and 380 mm above the workbench, using an A-weighted sound level meter. Background noise was recorded at the same location after the BSC fan was switched off and used for correction. The noise performance was considered qualified if the sound pressure level was 67 dB or lower.

**Illumination** was assessed on the work surface of the BSC along the centerline of the two interior sidewalls. Measurement points were spaced no more than 300 mm apart, with the minimum distance from any interior wall set at 150 mm. After switching on both the lighting system and blower fan, illuminance was measured sequentially at each point using an illuminance meter. The illumination performance was considered qualified if the average illuminance was 650 lx or above.

**Cleanliness** was assessed after the BSC had been operating for 10 minutes under normal working conditions. Particle counts were measured diagonally at a height of 200 mm above the workbench and at a distance of 100 mm from the interior surfaces or the front operating window. Cleanliness of the working area was required to comply with ISO Class 5 air cleanliness standards.

**Inflow velocity** was measured after opening the front operating window to the specified height. Airflow velocity was recorded using an anemometer at measurement points arranged in two horizontal rows at approximately 25% and 75% of the window height, with a spacing of 100 mm between adjacent points. The inflow velocity performance was considered qualified if inflow velocity was 0.50 m/s or above.

**Downflow velocity** was measured using an anemometer at positions 150 mm away from the interior walls or front operating window and 100 mm above the plane of the operating window. Measurement points were distributed in a square grid with 150 mm spacing. The downflow velocity performance was considered qualified if downflow velocity was ranged from 0.25 m/s to 0.50 m/s.

**HEPA filter integrity testing** included assessment of maximum leakage downstream of both the supply and exhaust HEPA filters. When the upstream aerosol concentration reached 20 μg/L, an aerosol photometer was used to scan the entire workbench surface at 20–30 mm downstream of the HEPA filter and the exhaust duct. The HEPA filter integrity performance was considered qualified if the maximum leakage was 0.01% or less for both supply and exhaust filters in Type-A BSCs, or if the maximum leakage was 0.01% or less for the supply filter and 0.005% or less for the exhaust filter in Type-B BSCs.

**Airflow smoke pattern** assessment included evaluation of downflow uniformity, airflow at the observation window, airflow along the edges of the front operating window, and the tightness of the active window. Smoke visualization was performed at predefined locations to assess airflow direction and stability. Airflow pattern performance was considered qualified if the airflow in the working area was uniformly downward, with no vortices, upward airflow, dead zones, or observable escape of air from the cabinet.

**References**

China Food and Drug Administration. (2011). YY0569-2011, Pharmaceutical industry standards of the People's Republic of China-Biosafety cabinet: Beijing: Standards Press of China.

General Administration of Quality Supervision, I. a. Q. o. t. P. s. R. o. C. (2014). Biological safety cabinet use and management specifications. Beijing: National Certification and Accreditation Supervisory Administration Committee.

Ministry of Construction of the People's Republic of China. (2005). JG170-2005, Industrial standards of the construction industry of the People's Republic of China-Biosafety cabinet: Beijing, Ministry of Construction of the People's Republic of China.
